# Three overlooked key functional classes for building up minimal synthetic cells

**DOI:** 10.1093/synbio/ysab010

**Published:** 2021-04-20

**Authors:** Antoine Danchin

**Affiliations:** Kodikos Labs/Stellate Therapeutics, Institut Cochin, Paris, France; School of Biomedical Sciences, Li KaShing Faculty of Medicine, Hong Kong University, Pokfulam, SAR Hong Kong, China

**Keywords:** mycoplasma, EF-P/EIF5A, Maxwell’s demon, EttA, ddhCTP

## Abstract

Assembly of minimal genomes revealed many genes encoding unknown functions. Three overlooked functional categories account for some of them. Cells are prone to make errors and age. As a first key function, discrimination between proper and changed entities is indispensable. Discrimination requires management of information, an authentic, yet abstract, currency of reality. For example proteins age, sometimes very fast. The cell must identify, then get rid of old proteins without destroying young ones. Implementing discrimination in cells leads to the second set of functions, usually ignored. Being abstract, information must nevertheless be embodied into material entities, with unavoidable idiosyncratic properties. This brings about novel unmet needs. Hence, the buildup of cells elicits specific but awkward material implementations, ‘kludges’ that become essential under particular settings, while difficult to identify. Finally, a third functional category characterizes the need for growth, with metabolic implementations allowing the cell to put together the growth of its cytoplasm, membranes, and genome, spanning different spatial dimensions. Solving this metabolic quandary, critical for engineering novel synthetic biology chassis, uncovered an unexpected role for CTP synthetase as the coordinator of nonhomothetic growth. Because a significant number of SynBio constructs aim at creating cell factories we expect that they will be attacked by viruses (it is not by chance that the function of the CRISPR system was identified in industrial settings). Substantiating the role of CTP, natural selection has dealt with this hurdle *via* synthesis of the antimetabolite 3′‐deoxy‐3′,4′‐didehydro‐CTP, recruited for antiviral immunity in all domains of life.

## Graphical Abstract

**Graphical Abstract:**
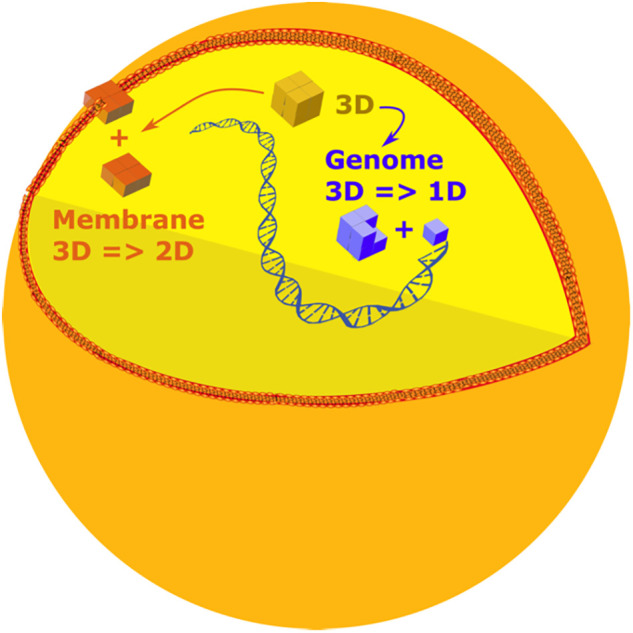

## 1.Introduction

Most studies meant to adopt a proper chassis for Synthetic Biology (SynBio) assume that metabolism progresses in a smooth and accident-free way. Yet, all physicochemical processes are prone to make errors and to age (1). Cells cope with metabolic accidents using maintenance functions that entail discrimination between correct and altered metabolites, so that they can get rid of wrong ones while preserving proper ones. Discrimination is an information-loaded process, an attribute of physics (2) seldom seriously considered in biology despite the omnipresent use of the word ‘information’ by biologists. Yet, if we accept that information is the currency of physics that best characterizes life, it cannot remain an abstract concept, we must see it embodied into material substrates. Another hidden difficulty associated with standard SynBio work involves two further categories of physics, space and time. While the cell’s machinery and expression of its program are explored in-depth to a great degree, the physicochemical limitations created by the need for growth are credited to a very narrow set of functions. Yet, growth is uniquely associated with life’s metabolism when cells give birth to a progeny. This condition is sometimes analyzed *via* smart efforts involving computational models (3), but genome-scale metabolic network modeling is generally overfitting, requiring continuous improvements (4). The importance of putting together the growth of a cytoplasm (three dimension), of a membrane (two dimensions) and of a genome (one dimension)—some sort of nonhomothetic growth (5)—should ask for metabolic coordination. Surprisingly, this fact, which should integrate together a significant number of biological functions likely unknown or unaccounted for, is consistently underestimated.

To make some sense of the vast number of unknown gene functions, we discuss here experimental elements that support their critical importance in information management and control of homeostasis when facing nonhomothetic growth. Finally, we emphasize that—contrary to a widespread belief—matter (mass) may have considerably more importance than energy for the construction of a cell. This is because the management of energy can be generic, can allow a large span of dissipative processes and has evolved in that way while preserving fitness. To be sure, instantiation of energy—yet another fairly abstract currency of reality—in life’s processes is illustrated in cells by the universal role of metastable phosphate bonds as its material implementation (6). ‘Better lose energy than control’ was a motto often used by Albert Szent-Györgyi, to highlight the material importance of the need for embodiment of information. Yet, in contrast to energy, the various implementations of material control systems rest on a fairly large number of components based on the cell’s building blocks. For example, these entities are made of nonrandom atoms, retained for their ability to generate stable covalent bonds at 300 K (the temperature of life) as well as their space-filling properties. This has unanticipated consequences. Matter (mass) has highly idiosyncratic properties, such as, for atoms again, covalent bond orientation, or, as an omnipresent feature of life processes, the ability to muster hydrogen bonds. Each one of these distinctive properties must be dealt with and somehow smoothed out. To avoid affecting negatively the functions they embody, this asks for the selection of solutions on a case by case basis. Material embodiment of abstract functions will then result in the widespread emergence of awkward solutions, ‘kludges’ that give life its ‘tinkering’ flavor. It is critical to understand that SynBio constructs cannot escape this particular snag.

## 2. Unusual functions for life

Since the publication of the short genome sequence of *Mycoplasma genitalium* in 1995, the key question of the biological relevance, then synthesis of genomes of minimal size was tackled in various laboratories (7). The rationale was that it should be possible to design a list of all genes necessary to construct the minimal genome of a cell able to multiply freely if provided with relevant nutrients. Studies involving bacteria from a variety of descents concurred to the identification of approximately 500 relevant genes. They encoded all the cell’s essential functions as well as the basic casing required for survival and multiplication when cells are supplied with all known metabolic building blocks (8). Yet, when comparing the cognate sequences of multiple organisms, it appeared progressively that no single gene coding for orthologous protein sequences was conserved across all (9). This illustrated that, while functions may be conserved, structures (and therefore sequences) are not. As a consequence, finding a sequence not related to any previously known sequence, does not mean that it is associated with an unknown function (10, 11). The effort culminated with the biochemical synthesis of the genome of *Mycoplasma mycoides*, strain Syn3.0.

This work reported the presence of 473 genes with the surprising announcement that 149 of those were of unknown function (12). First and foremost, this contention derived from structural—i.e. gene sequence—analyses. These analyses assumed that functions were ascribed based on a strong similarity principle. Yet, besides sequence biases linked to the highly specialized buildup of mycoplasmas, there is no one-to-one correspondence between the structure of a gene product and its biological function. This is vividly illustrated even in prevalent biochemical entities. As an example, the most abundant enzyme on the planet, utilized to fix carbon dioxide, Ribulose-Bisphosphate Carboxylase Oxygenase (RuBisCO, absent from mycoplasmas), has structural counterparts used as sulfur metabolic enzymes in completely different pathways (13). In contrast, the same activity may stem from multiple origins: nanoRNases, enzymes required in all cells—including minimal cells—stem from widely different structural descents (14). Less extreme examples can be found in the frequent recruitment of whole pathways for the development of ‘paralogous’ metabolism using enzyme paralogs [i.e. enzymes that have evolved to accommodate substrates related, but different, from their reference substrates (15)]. Finally, proteins endowed with enzyme activity may be recruited to display a physicochemical role foreign to their enzyme activity, such as the vitrous transition allowing transparency of the lens (16).

To highlight unforeseen yet key functions, we follow the symmetrical view, that we should first make a list of likely functions, and then look for structures that might embody them. Developing a list of necessary functions allowed the identification of a significant number of the genes deemed of unknown function in *M. mycoides* Syn3.0. Tenericutes are highly evolved organisms that lost many functions: this was the very reason for choosing members of this clade as chassis for SynBio. However, this very feature made that identification by structural similarity was problematic. To be sure, perfect adequacy between the recruited entities and their role in information management is not automatic and makes functional identification difficult. In addition, information is abstract and must be concretely embodied. Again, this results in the need for ‘kludges’, awkward but necessary solutions for an essential problem, in a way that varies depending on the organism of interest (17). Box 1 illustrates a few examples of how the transition from concepts to engineering could be implemented, with an attempt to constitute classes of kludges.

## 3. Focus on information-managing functions as critical to ‘animate’ biological chemistry

Life depends critically on mass-independent processes, i.e., on processes specifically requiring management of information (21). While this is widely accepted by physicists (2), the actual involvement of information as an authentic category of physics is hardly considered by most biologists. This is despite its life-like, ‘animated’, involvement in physics since the end of the XIXth century, when Maxwell proposed a concrete instantiation challenging the second law of thermodynamics: ‘If we conceive a being whose faculties are so sharpened that he can follow every molecule in its course, such a being, whose attributes are still as essentially finite as our own, would be able to do what is at present impossible to us. For we have seen that the molecules in a vessel full of air at uniform temperature are moving with velocities by no means uniform, though the mean velocity of any great number of them, arbitrarily selected, is almost exactly uniform. Now let us suppose that such a vessel is divided into two portions, A and B, by a division in which there is a small hole, and that a being, who can see the individual molecules, opens and closes this hole, so as to allow only the swifter molecules to pass from A to B, and only the slower ones to pass from B to A. He will thus, without the expenditure of work, raise the temperature of B and lower that of A, in contradiction to the second law of thermodynamics*’* (22). This particular agent was subsequently named after its author, a Maxwell's demon. To manage information in the omnipresent critical process of discrimination between classes of entities, we expect that cells code for proteins that operate as Maxwell's demons able to sort similar entities into relevant classes (23).

The embodiments of these fictitious agents display highly specific families of targets or are located spatially at precise sites. They are used to discriminate between classes of objects such as young and aged entities and drive them to a specific location or degradation processes for example. Remarkably, this view, currently forgotten, was already considered by Charles Sherrington very early on, before the discovery of the double helix: ‘We seem to watch battalions of specific catalysts, like Maxwell's “demons” lined up, each waiting, stop-watch in hand, for its moment to play the part assigned to it, a step in one or other great thousand-linked chain process. Yet each and every step is understandable chemistry […] In this great company, along with the stop-watches run dials telling how confrères and their substrates are getting on, so that at zero time each takes its turn’ (24). A general example of Maxwell’s demon’s like discrimination is offered by the multidrug resistance exporters that export foreign compounds while keeping authentic metabolites within the cell. These transporters use energy in the process. It can be strongly argued that a sizable amount of that energy dissipation is used for discrimination (25). As a general rule, it remains critical to explore in-depth all genes coding for proteins with a nucleotide-binding site which would predict an expletive dissipation of energy. This cataloguing is expected to highlight the presence of an information-managing process.

Proteins long annotated wrongly as ABC transporters, such as EttA in Bacteria (26, 27) or ABCE1 in Eukarya (28) are cases in point. Long mistaken for a membrane-associated ABC transporter, EttA is in fact a cytoplasmic protein. It helps ribosomes to select a specific family of mRNAs under limiting energy availability (29) and discriminating against the majority, using ATP in the process. ABCE1 plays a similar role in eukaryotic cells. Interestingly, it belongs to a complex with protein Nsp1 of SARS-CoV-2, presumably as a way to make the mRNA-like genome of the virus the preferred translated substrate in host cells (30). There is a further considerable number of GTP- or ATP-hydrolyzing proteins that do not have a well-established function. Many are required for shaping ribosomes into their proper functional form, discriminating against erroneous shapes. They are conserved in the minimal set of functions that is required for life (17). Curiously, they have seldom been explicitly input in reflections about SynBio minimal constructs.

**Box 1:** Illustration of concrete examples of the three categories of functions generally overlooked in synthetic biology chassis

The synthetic genome of *Mycoplasma mycoides* Syn3.0 is a prototype for future constructs of streamlined bacterial chassis (12). When first published, it listed 149 genes of unknown function. Some important families were identified, as listed in the table at the end of this box. The most elusive family—illustrated severe times in the genome of Syn3.0—was that of ‘kludges’ used to resolve the inadequacy between an abstract function and its concrete embodiment. A kludge is, by definition, an anecdote and thus intrinsically resistant to easy classification. It may however be useful to collect relevant anecdotes and cluster them into various families overlapping the general functional families that make a living cell. Kludges will appear each time a novel function emerges in a particular organism. This is because no preexisting design would make the entity retained to embody the function finely adapted to its new host. The corresponding process is often revealed as ‘convergent evolution’. As an example, the function of nanoRNases—cleaning up RNA debris following RNA degradation—provides an interesting illustration of a kludge family (MMSYN1_0139). At least three different structural descents have been recruited to fulfill the function, and some are sometimes present simultaneously in the same organism (e.g. NrnA and NrnB in *Bacillus subtilis*). Another activity, displayed by protein MMSYN1_0283, is likely to remove accidental ribonucleotides in DNA. As with the function of nanoRNAses—belonging to at least three phylogenetic descents (18)—this function is a conserved requirement likely to be embodied in a variety of protein structures. Consequences of horizontal gene transfer are also likely to result in the emergence of classes of kludges. The newly acquired genes code for functions that were well adapted to a source organism but are unlikely to be immediately running smoothly in the new host. Many unknown functions present in pathogenicity islands are likely to pertain to this class. Exploring the *Pseudomonas putida* KT2440 chassis to explain the puzzling disruption of an essential gene, *tmk*, by a long insert encoding many genes of unknown function (19), might reveal cases in point. Another class, also highly relevant for SynBio constructs, is the adaptation of a new building block to a recipient host. This is the situation created, for example, when a new amino acid such as selenocysteine is introduced in the translation repertoire. Specific translation factors and RNA structures, for example, are required to minimize mistaken input at wrong sites. Early in evolution this must have been illustrated by the role of proline as a variant of a chemically well-defined kind, that of ‘amino acids’. Indeed, using a secondary amine in protein synthesis imposed the use of *ad hoc* biochemical solutions. Proteins with runs of proline residues require a whole translation factor EF-P-related set of genes (not only EF-P itself, MMSYN1_0391, but also its post-translational modification, e.g., MMSYN1_0697). Subsequently, specific peptidases were also needed to clean up peptides resulting from interrupted translation at peptides ending with a proline residue (e.g. MMSYN1_0305 in Syn3.0). This handicap was so strong that conservation of proline repeats in proteins (…PP…, …PPP…, etc., note that, for a gene with easily identified function, a PPP triplet is widely conserved in valyl-tRNA synthetase MMSYN1_0260, for example) could be used to validate in Syn3.0 sequence alignments that displayed only very limited similarity (17). Finally, there is an infinite variety of highly specific roles associated with the shaping and assembly of complex structures or shapes that need to be stabilized, perhaps not in the short term, but in the long term. This is illustrated by protease MMSYN1_0500, which cleaves off the first nine residues of ribosomal protein L27 from a form used for maturation of the 50S ribosomal subunit, to a form functional in translation. Implementing this kludge may be necessary when SynBio engineers prefer to use a chassis related to Firmicutes rather than other bacterial clades. Proteins stabilizing unusual 3D structures in ribosomal RNA are also critical (e.g. MMSYN1_0298, MMSYN1_0299). Finally, this may account for a large number of ‘orphan’ proteins such as the possible ‘gluons’ rich in aromatic residues and observed in many genomes (20). The second functional family explored here in details pertains to ‘discrimination’. This process is linked to dissipation of energy and involved in transport of specific substrates (specific metals, polyamines, peptides etc.), proteins and RNA maintenance (e.g. MMSYN1_0410), as well as specific processes such as cell division (this is the case of FtsH, MMSYN1_0039, for example). The third functional family illustrates coordination of nonhomothetic growth. As discussed in the text, it accounts for the presence of PyrG, CTP synthetase (MMSYN1_0129) in Syn3.0. The role of CTP is subsequently more difficult to pinpoint, but it certainly critical for nucleic acid synthesis in transcription and replication, as well as in translation (tRNA synthesis). Nine examples of novel routes for engineering cells following the concepts developed in the text. PubMed references are indicative. Besides NTP/polyphosphate-dependent energy sources involved in information management, experiments using the methylation tag as an energy-dependent discrimination tag are also suggested. A Maxwell’s demon is an agent—generally a protein—that dissipates energy in order to discriminate between classes of entities, specific energy states or specific locations. An example is discrimination between old and young proteins, triggering degradation of the old ones without affecting the young ones see text for further description of the role of a Maxwell’s demon.			

	Concept	Engineering synthetic cells	References PubMed Id

Discrimination	Maxwell’s demons	Elimination of aged proteins, *via* management of isoaspartate residues Discrimination of mRNA classes by different EttA-like proteins Lon activity during stationary phase of growth	30578298 30597160 32366590
Material implementation	Embodiment of abstract information into concrete matter resulting in ‘kludges’	Elimination of the requirement for factor EF-P Changing aspartate residue in bacterial ribosomal protein S11 Bartonella as a new chassis: replacing a nanoRNase class by another class in synthetic constructs	31237868 10217780 22262096
1D 2D 3D homeostasis	Nonhomothetic growth	Regulation of the 3D structure of CTP synthetase complexes Design of a synthetic cytosine phosphoribosyltransferase and evolution Implementing viperin synthesis in synthetic cells	31431504 16152602 32937646

## 4. Coordinating nonhomothetic growth

This information managing process is sometimes associated with an unexpected source of energy, for example, in the case of chromosome partition in *Bacillus subtilis,* hydrolysis of the nucleotide cytidine triphosphate (CTP) instead of the expected ATP (31). Remarkably, this is not an accident. To be sure, this feature, which should be regarded as central in SynBio constructs, reveals how natural selection succeeded in solving the problem of nonhomothetic growth when cells, the atoms of life, are poised to grow at some point in their destiny. It highlights the metabolic processes that, almost always, are organized in such a way as to allow cell growth as soon as the opportunity to multiply arises. In our usual physical space, growing introduces an inevitable constraint. The cell must put together the growth of its cytoplasm (3D, therefore), that of the membrane surrounding it (2D) and that of its genome (1D, because nucleic acids are linear polymers). This creates an enormous metabolic pressure to make ‘too much’ of the membrane, and even greater pressure to make ‘too much’ of the genome. The way these physical discordancies have evolved into cellular harmony needs to be unraveled, possibly investigating the origins of the first cells.

Many convincing scenarios suggest that life developed from a primitive metabolism in several stages over more than 3.5 billion years. As a consequence, one might have feared that each organism had found individual idiosyncratic solutions to this constraint. For example, a key module of the cell’s metabolism, carbon central metabolism (CCM), was demonstrated to be connected to DNA replication initiation and elongation in *B. subtilis*. This connection postulated control of DNA synthesis by post-translational modification or allosteric regulation involving the CCM (32). It did not entail nonhomothetic growth. Nevertheless, a coupling between the CCM and DNA synthesis is certainly contributing to help the cell solve this hurdle, possibly in a fairly general manner. Amazingly however, a generic metabolic pathway deployed in the cytoplasm produces the building materials of all three major components. It happens that a single cell building block, CTP, was used for this purpose in all organisms (with the usual reservation when investigating life forms that there is a good chance that we will discover somewhere an exception to any rule).

**Figure 1.**
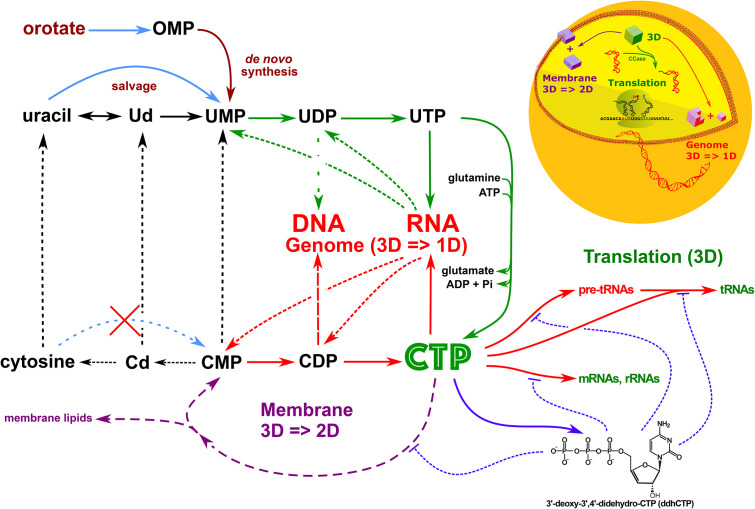
Nonhomothetic growth in a spherical cell. Pyrimidine biosynthesis begins with orotate and goes through uracil derivatives (brown arrow). Dotted arrows label catabolic pathways. Synthesis of CTP (in green) coordinates the growth of the cytoplasm (*via* RNA synthesis and tRNA CCA transferase), the growth of membranes (*via* cytosine-containing membrane lipid precursors, purple large dash arrows) and synthesis of the genome (*via* RNA degradation-dependent DNA synthesis, red arrows). Existing phosphoribosyltransferase activities are shown with light blue arrows. It is remarkable that cytosine phosphoribosyltransferase does not exist in identified organisms (red cross on light blue dashed line) while other pyrimidine phosphoribosyltransferases (orotate and uracil) are commonplace (light blue arrows). The catabolism (black dotted arrows) or salvage (black arrows) of pyrimidines cycles *via* uracil derivatives. The purple arrow coming from CTP shows viperin-mediated synthesis of 3′‐deoxy‐3′,4′‐didehydro‐CTP (ddhCTP), illustrating the antifunction that counteracts control *via* CTP synthetase. Dotted purple stopped lines indicate where ddhCTP blocks CTP usage.

Several observations, directly impacting SynBio, substantiate this view. Minimal cells are grown on media supplemented with all necessary building blocks to dispense having them to carry over the corresponding biosynthetic pathways. Direct input of nucleoside polyphosphates is excluded because these metabolites cannot permeate cells with their strongly negative intracellular electric potential in the absence of specific transporters. However, the presence of nucleoside kinases, nucleoside diphosphokinases, and heterocyclic base phosphoribosyl transferases allows synthetic cells to build up a near-complete NTP complement when heterocyclic bases or nucleosides are present in the medium (uracil phosphoribosyl transferase, MMSYN1_0798, is indeed present in the minimal Syn3.0 setup). Yet, CTP is an exception: cytosine phosphoribosyltranferase has not been identified in any extant organism (30), and cytosine-derived molecules are systematically recycled to uracil-derived metabolites. This explains why including cytosine in growth media cannot make CTP synthetase (PyrG) dispensable (Figure 1). As a consequence, PyrG is an essential enzyme in all minimal constructs (12). This observation is in line with the role of CTP as a unique regulator of nonhomothetic growth. Instead of being used in most steps involved in sorting and recovering energy, as performed by purine nucleoside triphosphates, pyrimidine nucleoside triphosphates are also involved in a narrow set of functional families. This is even more extreme for CTP. CTP is not only involved in RNA synthesis as are the other NTPs. It is also uniquely required for the generation of membranes, maturation and repair of transfer RNAs (*via* the addition of their CCA terminal end), synthesis of coenzyme A and even, in eukaryotes, formation of the membrane-embedded dolichyl-phosphate required for glycosylation of proteins. Finally, as we have seen, a further coupling between CTP synthesis and DNA replication has been identified in the process of DNA segregation, which is CTP- not ATP-dependent (33). PyrG itself must therefore be the enzyme coordinating nonhomothetic growth. Interestingly, nonhomothetic growth may benefit virus multiplication by allowing unchecked genome multiplication. A number of SynBio endeavors aim to create cell factories that will be implemented in natural environments. We must expect that they will be attacked by viruses (it is not by chance that the function of the CRISPR system was identified in an industrial setup). It is remarkable that natural selection opened up an ‘antifunction’ used to interfere with the critical role of CTP. An antiviral innate immunity response developed in all domains of life with the emergence of viperins (30, 34), enzymes synthesizing the CTP antimetabolite 3′‐deoxy‐3′,4′‐didehydro‐CTP (ddhCTP, Figure 1).

At this point of our exploration, we should try and understand the unwanted consequences of embodiment of information into the matter made of atoms and molecules, with their idiosyncratic properties that have no intrinsic reason to be designed for information-related functions. How do cells adjust to this constraint?

## 5. Awkward but necessary solutions to critical functional problems

It is commonplace to remark that the way biological entities are put together is akin to tinkering, leading to the extraordinary apparently gratuitous diversity of life forms. Yet, engineers know that when they make extremely elaborate constructions they often have to give them a final touch with some kind of ‘kludge’ or ‘patch’, i.e. an extra component that looks awkward and has not any decent design, but which is necessary to allow the construct to behave properly. There is an unlimited number of such kludges in living organisms. Knowing this can be used to uncover a wealth of unknown functions (details are provided in Box 1).

**Figure 2.**
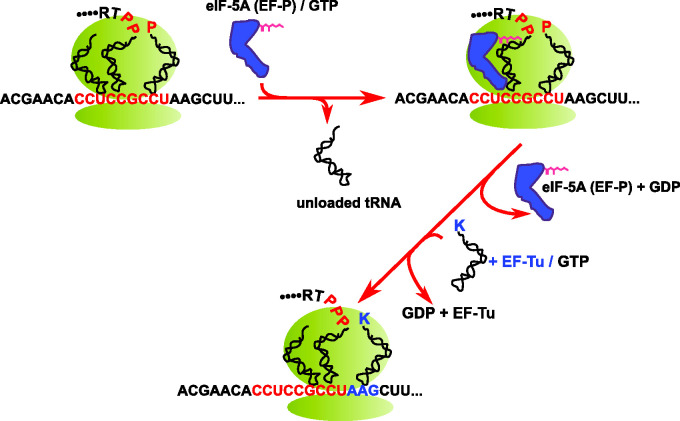
An awkward but key function, proline input in polypeptides. Here, we illustrate the complex setup that allows input of proline, not an amino acid, into proteins. A mRNA with three consecutive proline codons is read by a ribosome (dark green). The first CCU codon has been deciphered. The corresponding unloaded tRNA is in the exit (E) ribosome site. The second proline codon (CCG), which has been input in the nascent polypeptide, carried by a tRNA_pro_ loaded by the polypeptide in the P site is ready to accept a third proline residue. However, input of proline has slowed down considerably the formation of a peptide bond. This leaves time for factor EF-P (dark blue), loaded with GTP and with its important lysine post-translational modification (in red) to take the place of the tRNA exiting the E site. EF-P recognizes the proline-loaded tRNA interacting with the third CCU codon and helps formation of the proline–proline peptidyl link. Subsequently, it is released from the ribosome with concomitant GTP hydrolysis and entry of the next EF-Tu-bound loaded tRNA (here a tRNA deciphering a lysine codon, AAG). Elongation factor EF-P/EIF5A is necessary to speed up proline input during translation, in particular when several proline residues belong to the polypeptide. Not only does this function require a specific protein, but the protein must be further modified, at a considerable genetic cost. The corresponding modification is yet another example of a ‘kludge’ as it differs in different organisms.

Most kludges are species-specific, but some, discovered during the early days of life, are more general (17). For example, to improve our inferences in the identification of unknown functions, we pointed out a still poorly documented but universal process required for protein biosynthesis. We noticed that the proline residue in not an amino acid but a cyclic secondary amine, not fit to use the standard translation system. Indeed, introducing proline residues into proteins imposed the implementation of an *ad hoc* translation elongation factor, EF-P/EIF5A (35). This has a high genetic cost with complex, species-dependent, modification at a key lysine/arginine residue (36), a likely source of still unknown unique *ad hoc* functions embodied in different species (Figure 2). Here is a second example, with a narrower scope. The process of ribosome synthesis and assembly displays different features in different organisms, depending on the structure/sequence of their ribosomal RNA. In the Firmicutes clade, two forms of ribosomal protein L27 are used. The first one is needed for assembly of the large subunit. A second, shorter form is used during the process of translation. The latter is the result of a highly specific proteolysis that removes the first nine amino acids of the initial polypeptide. The corresponding protease RppA/YsxB is encoded in a gene which is only present in Firmicutes (37). RppA belonged therefore to the essential unknown genes of the *M. mycoides* Syn3.0 construct (17). Another, more sophisticated example is the need for the cell to cope with inevitable metabolic accidents. This is illustrated by accidental generation of dehydroalanine (iminopropionate) in many processes such as those involving synthesis, salvage, and racemization of serine or cysteine. This molecule is toxic and cells have evolved a catalog of proteins of the RidA family to inactivate it (38). Finally, we have already alluded to the function of nanoRNases, essential enzymes needed during mRNA decay which leaves behind 2- to 5-nt-long oligoribonucleotides, termed ‘nanoRNAs’. They belong to at least three different structural lineages (39). We can expect that many similar cases will be uncovered progressively, accounting for the extraordinary diversity of life forms.

## 6. Will we bring ‘animation’ to synthetic biology?

To improve our successes in SynBio, we suggested that we must put significant effort on understanding the paths for the management of information that ‘animates’ chemical biology. We also noticed that we must also make explicit in metabolic terms the physical constraints associated with the need for growth. This revealed an unexpected role of the CTP nucleotide synthesis. Identification of this metabolic omnipresent bottleneck should for example lead us **(**Box 1**)** to investigate the way CTP synthetase is organized into the structures named ‘cytoophidia’ as important structures for minimal cells (40). We should also monitor functionally related genes for novel chemical pathways, such as the remarkable ones which led to the synthesis of nucleoside triphosphate analogs as antiviral metabolites (34).

To make a cell factory, we must go well beyond these minimal requirements. Besides the construction of a minimal cell, exploring the vast domain of biological chemistry opens up an unlimited number of unknown functions. In a minimal autotrophic organism, at least 1000 genes code for proteins that make the cell’s casing more resistant and develop metabolic pathways that generate all building blocks from minerals or a limited set of basic organic compounds. Functions required for synthesis and turnover of building blocks are distributed along with an unlimited panel of functions, many of which certainly still remain unknown. The same is true for restricted-energy management. Also, while we took growth into account, our overview did not consider that cells must keep generating a progeny for many generations. It did not consider either the fact that they are doomed to interact with other cells, be it only during the very process of generating a progeny. Recruiting structures to build up a predator, for example, will trigger an explosion of functions meant to escape predation. Because cells never exist in isolation (creating a progeny is a case in point), the number of functions coping with the management of populations is entirely open. This is witnessed for example with functions relating to the predator/prey challenge, opening up an endless set of functions, including ‘mild’ resolution of the conflict, such as mutualism or commensalism. Novel functions in this domain keep being discovered (34, 41). To be sure, the very concept of function is open-ended. Functional analysis tells us that each time a function appears, it will open up for further functions to emerge, be it only as the function negating the function of interest. The emergence of novel function is the rule rather than the exception. Overall, this means that we cannot expect that the list of genes with the unknown function will shrink any time soon. This is particularly true for metabolic functions involving highly specialized pathways using the infinite variety of chemicals that keep accumulating on the surface of the Earth. We can expect this to be an open source for novel metabolic engineering.
